# Effects of acupuncture combined with strength training and whole-body vibration training on patients with chronic ankle instability: a randomised controlled trial

**DOI:** 10.3389/fbioe.2025.1615121

**Published:** 2025-08-08

**Authors:** Jian He, Shuwan Chang, Heng Liu

**Affiliations:** ^1^ BAYI Orthopedic Hospital, China RongTong Medical Healthcare Group Co. Ltd., Chengdu, China; ^2^ Department of Sports and Human Science, Sichuan Sports College, Chengdu, China; ^3^ College of Physical Education, Chongqing University, Chongqing, China

**Keywords:** chronic ankle instability, acupuncture, strength training, whole-body vibration training, balance ability, ankle rehabilitation

## Abstract

**Objective:**

This study aims to investigate the rehabilitative effects of adding whole-body vibration training (WBVT) on the ankle function, muscle strength, and balance ability of patients with chronic ankle instability (CAI) based on strength training combined with acupuncture.

**Methods:**

A total of 49 university students with unilateral CAI was divided into an experimental group (strength training + acupuncture + WBVT, n = 25) and a control group (strength training + acupuncture, n = 24). The intervention lasted for 8 weeks (3 times per week). The evaluation indicators included cumberland ankle instability tool (CAIT), foot and ankle ability measure (FAAM), isokinetic muscle strength, proprioception, and balance ability.

**Results:**

1) At the 8th week, intergroup comparison showed that the experimental group had higher FAAM-ADL (*p* = 0.043) and peak plantar flexion torque of the ankle (*p* = 0.020) than the control group. The anterior-posterior displacement (*p* = 0.002) and average speed (p = 0.046) were lower than those in the control group. 2) At the 8th week compared with baseline, within-group comparison showed that in the experimental group, CAIT, FAAM-ADL, and FAAM-Sport increased by 16.2, 36.2, and 37.8% respectively (*p* < 0.01). The peak torque of ankle dorsiflexion, plantar flexion, inversion, and eversion increased by 34.2, 19.4, 12.3, and 12.3% respectively (*p* < 0.05). The threshold of motion perception of ankle plantar flexion decreased by 15.1%. The anterior-posterior and medial-lateral displacements decreased by 22.2% and 17.5% respectively, and the average speed decreased by 31.2% and 13.8% respectively (*p* < 0.05). In the control group, FAAM-ADL and FAAM-Sport increased by 20.9% and 26.3% respectively (*p* < 0.01). The anterior-posterior and medial-lateral displacements decreased by 10.9% and 12.5% respectively, and the average speed decreased by 18.9% and 9.2% respectively (*p* < 0.05).

**Conclusion:**

Adding WBVT to strength training and acupuncture significantly improves ankle function, plantar flexion strength, and anterior-posterior balance in CAI patients, supporting its integration into comprehensive, non-surgical rehabilitation protocols.

## 1 Introduction

Chronic Ankle Instability (CAI) is a condition resulting from inadequate or delayed treatment of ankle sprains, characterized by recurrent sprains, persistent pain, joint weakness, instability, and restricted movement (Lal et al., 2023). The pathological basis lies in the injury and poor repair of the soft tissues, muscles, and joint capsules around the ankle joint, which leads to decreased joint stability (Koshino and Kobayashi, 2023). It is also associated with proprioceptive disorders (Xue et al., 2021), muscle strength imbalance (Khalaj et al., 2020), and decreased balance ability (Mollà et al., 2021). CAI not only affects the physical function of patients but may also lead to chronic changes such as ankle cartilage degeneration and traumatic arthritis (Kim et al., 2020), which severely impact the quality of life and athletic ability. Current treatments for CAI face many challenges. Although surgical treatment can solve some problems, it has limitations such as invasiveness and long recovery time (Sanhudo et al., 2023). Non-surgical treatments are relatively safe, but traditional methods have limited effectiveness and longer treatment periods (Tsikopoulos et al., 2018). Therefore, optimising non-surgical treatment plans and exploring more effective rehabilitation methods are of great clinical significance.

Acupuncture (Zhuang et al., 2013), a traditional Chinese medical treatment, has demonstrated promising benefits in CAI rehabilitation. By stimulating specific acupoints, it can effectively relieve pain (Huang et al., 2023), improve ankle proprioception (Yan et al., 2013), increase ankle mobility (Mullins et al., 2021), and promote tissue repair (Park et al., 2013). Combined with modern rehabilitation concepts, the combination of acupuncture and strength training may have a synergistic effect and further enhance the therapeutic effect. Our previous study showed that adding acupuncture to 8 weeks of strength training for patients with CAI can further improve the anterior-posterior balance ability, ankle dorsiflexion and plantar flexion strength, and eversion proprioception (Chang et al., 2024).

It is worth noting that Whole-Body Vibration Training (WBVT) (Cheng et al., 2022), as an effective rehabilitation method, can effectively activate the neuromuscular system by transmitting vibratory stimuli to the body (Van Heuvelen et al., 2021), enhance muscle strength (Cheng et al., 2025), and improve balance ability (Asahina et al., 2023). In addition, WBVT has positive effects on improving the balance ability (Sierra et al., 2018), muscle strength (Hoveidaei et al., 2024), and proprioception (Chang et al., 2021) of individuals with CAI. Moreover, the combination of WBVT with other rehabilitation methods (such as balance training) (Coelho-Oliveira et al., 2023) can further enhance the therapeutic effect of CAI. To our knowledge, there is no literature reporting the therapeutic effects of strength training + acupuncture + WBVT on patients with CAI. While previous studies have explored individual effects of acupuncture and WBVT, their combined impact within a comprehensive rehabilitation protocol remains under-investigated.

To fill the gap in current research, based on our previous study (Chang et al., 2024), this study used strength training combined with acupuncture as the control group and strength training combined with acupuncture + WBVT as the experimental group to further investigate the rehabilitative effects of the combined intervention (strength training + acupuncture + WBVT) on CAI. This study aims to provide references for exploring effective rehabilitation plans for CAI. The research hypothesis is that adding WBVT intervention to strength training combined with acupuncture can further improve the stability and function of the ankle joint, balance ability, proprioception, and muscle strength of patients with CAI.

## 2 Participants and methods

This study was approved by the Ethics Committee for Human Trials at Sichuan Sports College (CSSC2024.7). Based on our previous research findings on CAI interventions (Chang et al., 2024), and considering the experimental design of this study (2 groups × 2 measurements) as well as the anticipated 10% dropout rate, the sample size was calculated using the G-power software. With an effect size set at 0.3, a power of 0.8, and a significance level α of 0.05, the minimum required sample size was determined to be 44 participants, who were university students with unilateral CAI.

Inclusion Criteria (Chang et al., 2024): aged between 18 and 25 years; passed a health check-up with no other significant diseases; met the CAI criteria assessed by the Cumberland Ankle Instability Tool (Lal et al., 2023), including at least one severe ankle sprain experience within the past 12 months, accompanied by pain, swelling, and other inflammatory reactions, and affecting daily activities for at least 1 day; at least two episodes of giving way, spraining, or a feeling of instability in the unilateral ankle within the past year; a cumberland ankle instability tool (CAIT) score below 24; participants were aware of the study content and provided informed consent. The study adhered to the principles of the Helsinki Declaration.

Exclusion Criteria (Chang et al., 2024): foot deformities or abnormal gait; history of lower limb trauma; suffering from movement disorders, epilepsy, or cardiovascular diseases; positive results in the anterior drawer test or talar tilt test of the ankle, excluding patients with structural ankle instability.

Participants were randomly assigned to groups using a digital random allocation method. First, each participant was assigned a unique number, and random numbers were generated using a computer. Subsequently, participants were sorted according to the random numbers and allocated to the experimental and control groups in sequence based on their numbers. Participants with odd numbers were assigned to the experimental group, while those with even numbers were assigned to the control group. If the total number of participants was odd, the last participant was randomly assigned to one of the groups to ensure balanced numbers. Due to the nature of the interventions (e.g., acupuncture and WBVT procedures), blinding participants and assessors was challenging. Thus, this study did not employ blinding. During the study, if participants withdrew for personal reasons, new participants were recruited from a standby list, which was also determined through random number generation and sorting. Ultimately, the experimental group consisted of 25 participants (18 males and 7 females), and the control group had 24 participants (17 males and 7 females). There were no statistically significant differences between the two groups in terms of age, height, weight, ankle stability and function, isokinetic muscle strength and proprioception, and balance ability (P > 0.05), ensuring the comparability of baseline characteristics (Table 1).

**TABLE 1:** Comparison of baseline data between the two groups.

Index	Pattern	Experience group (*n* = 25)	Control group (*n* = 24)	p
Age (y)		20.3 ± 1.7	20.6 ± 2.0	0.634
Stature (cm)		173.6 ± 4.6	172.8 ± 5.0	0.499
Body mass (kg)		66.5 ± 6.8	65.9 ± 7.2	0.730
N (Male/Female)		18/7	17/7	
CAIT (Scores)		18.5 ± 3.5	18.8 ± 4.2	0.779
FAAM-ADL (Scores)		42.5 ± 9.8	43.0 ± 8.6	0.759
FAAM-Sport (Scores)		13.5 ± 3.2	13.7 ± 3.3	0.876
Ankle 60°/s peak torque (Nm)	Dorsiflexion	14.3 ± 3.5	13.6 ± 4.4	0.542
	Plantar Flexion	67.0 ± 11.2	65.6 ± 13.0	0.658
	Inversion	30.0 ± 7.9	29.2 ± 6.5	0.865
	Eversion	28.5 ± 5.3	27.8 ± 5.9	0.686
Ankle Kinesthesia (°)	Dorsiflexion	2.10 ± 0.65	2.13 ± 0.70	0.923
	Plantar Flexion	2.72 ± 0.70	2.68 ± 0.70	0.840
	Inversion	2.85 ± 0.43	2.90 ± 0.50	0.696
	Eversion	3.01 ± 0.53	2.97 ± 0.48	0.755
Displacement (mm)	Anterior-posterior	67.6 ± 11.0	68.2 ± 12.1	0.853
	Medial lateral	59.0 ± 10.1	60.2 ± 9.6	0.561
Average speed(mm/s)	Anterior-posterior	14.1 ± 3.6	14.3 ± 2.9	0.909
	Medial lateral	12.3 ± 2.7	12.0 ± 2.2	0.834

Note: CAIT, cumberland ankle instability tool; FAAM-ADL, Foot and Ankle Ability Measure - Activities of Daily Living Scale; FAAM-Sport, Foot and Ankle Ability Measure - Sport scale.

### 2.1 Intervention protocol

Initially, both the control and experimental groups underwent the same strength training + acupuncture intervention as in our previous study (Chang et al., 2024). On this basis, the experimental group received WBVT intervention. The intervention lasted for 8 weeks, with 3 sessions per week. All participants maintained their original lifestyle and were visited weekly by researchers either in person or via phone to record their living conditions.

**FIGURE 1:**
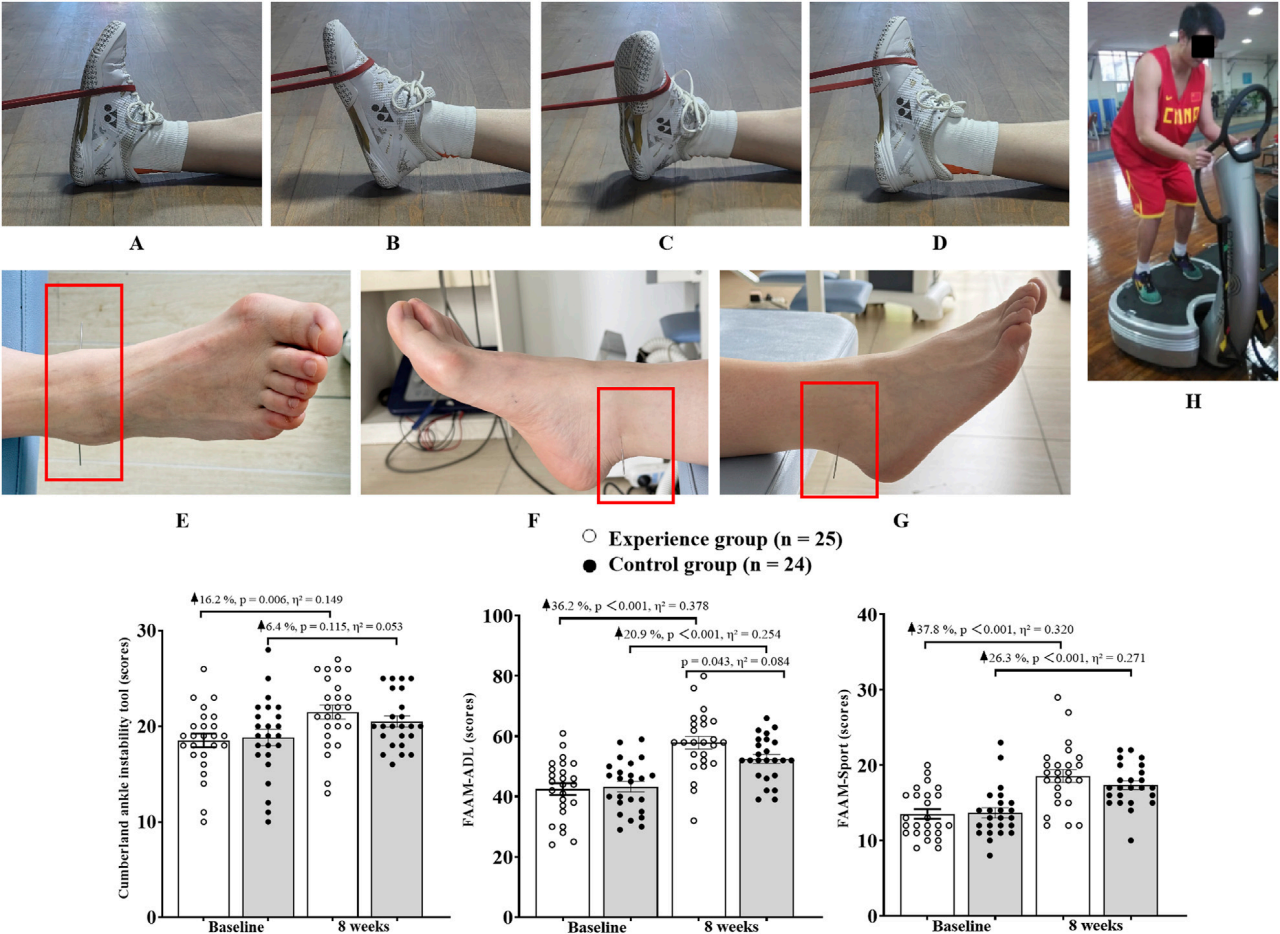
Participants were involved in ankle stability and function testing. Note: FAAM-ADL, Foot and Ankle Ability Measure - Activities of Daily Living Scale; FAAM-Sport, Foot and Ankle Ability Measure - Sport scale. **(A)** Ankle dorsiflexion training; **(B)** Ankle plantar flexion training; **(C)** Ankle inversion training; **(D)** Ankle eversion training; **(E)** Acupuncture points location; **(F)** Taixi (KI3); **(G)** Kunlun (BL60); **(H)** Example of WBVT platform use.

Strength Training: Participants performed resistance training for the ankle using elastic bands (Brand: China Anta. Color: Red. Specifications: 2080 × 4.5 × 32 mm. Resistance: 15–35 pounds). The training included four movements: dorsiflexion, plantar flexion, inversion and eversion (Figures 1A–D). Each movement was completed in 5 sets, with 10–15 repetitions per set and a rest of 15–20 s between sets. A rest of 60 s was taken between the four movements (Chang et al., 2024).

Acupuncture Treatment: Acupuncture was performed at two acupoints (Figures 1E) of the ankle: Taixi (Figures 1F, KI3, located at the depression to the back of the medial malleolus and between the Achilles tendon. This acupoint promotes blood circulation, alleviates foot congestion, provides analgesia, and treats medial malleolus injuries (Chang et al., 2024)) and Kunlun (Figures 1G, BL60, located at the depression to the back of the lateral malleolus and between the Achilles tendon. This acupoint has an analgesic effect and can treat lateral malleolus injuries (Chang et al., 2024)). Sterile stainless steel needles (Specification: 0.25 × 40 mm) were used, with a vertical insertion depth of 10–15 mm and a duration of 10–15 min per session (Chang et al., 2024).

WBVT Intervention: WBVT was combined with progressive load adjustment (Van Heuvelen et al., 2021; Li et al., 2019). Whole-body mechanical vibration was generated by a commercially available vibrating platform (Power Plate^®^ my7™, Performance Health Systems, Northbrook, IL, United States). The device uses two synchronized motors to deliver tri-planar (vertical, anterior-posterior, and medial-lateral) sinusoidal mechanical vibration at frequencies adjustable from 25 Hz to 50 Hz and amplitudes of 2 mm (low) or 4 mm (high). All vibration parameters were verified with a calibrated tri-axial accelerometer prior to the study to ensure accuracy and reproducibility. The 8-week WBVT intervention included four progressive stages, with vibration amplitudes increasing from 2 mm (weeks 1–4) to 4 mm (weeks 5–8). For the first 2 weeks, participants adapted to bipedal standing at 30 Hz. In weeks 3–4, they transitioned to unilateral standing (on the affected side) at 35 Hz. In weeks 5–6, the frequency was increased to 40 Hz with the addition of balance challenges with eyes closed. In weeks 7–8, the 40 Hz vibration was combined with a 1 kg load to enhance neuromuscular control. Each training session included 3 sets × 60 s of vibration, with a rest of 60 s between sets (Van Heuvelen et al., 2021).

Exercise Compliance Control: Rehabilitation therapists supervised the participants’ postures and recorded any adverse reactions throughout the process to ensure the safety and standardisation of the training. To ensure the quality of the intervention, all training sessions were conducted in the laboratory and triple compliance management was implemented: researchers supervised on-site and filled in standardised record sheets; participants provided daily feedback on training completion and subjective feelings through electronic logs; a compliance standard of ≥80% attendance (19/24 sessions) was set. Those who did not meet this standard were considered dropouts. Weekly training reminders were sent, and monthly progress feedback meetings were held to maintain participant engagement through dynamic communication.

Sample Dropout Criteria: Voluntary withdrawal, training-related adverse events (pain score ≥6), insufficient compliance (absent for three consecutive sessions or total completion rate <50%), and loss to follow-up (uncontactable for over 2 weeks). For dropouts, the last available data were recorded, and standby participants were recruited according to the initial random allocation principle to ensure the sample size met the statistical requirements.

### 2.2 Ankle stability and function tests

The CAIT was used to assess the stability of the participants’ foot-ankle complex. Designed by Hille et al. (2006), the CAIT consists of nine questions for diagnosing and grading ankle instability. Both ankles were scored separately, with each question assigned different points based on the number of options. The maximum score for a single ankle is 30 points. The criterion for unilateral ankle instability is ≤24 points. A higher score indicates better foot-ankle stability. The CAIT has been proven to have good test-retest reliability (ICC = 0.930) and internal consistency (Cronbach’s alpha = 0.845–0.878) among Chinese people (Zhu et al., 2012).

The Foot and Ankle Ability Measure (FAAM) was used to evaluate the participants’ ankle function. The FAAM consists of two subscales: the Activities of Daily Living scale (FAAM-ADL) and the Sports scale (FAAM-Sport). The FAAM-ADL includes 21 questions with a total score of 84 points, while the FAAM-Sport includes eight items with a total score of 32 points. A higher FAAM score indicates better ankle function (Sierevelt et al., 2018). The FAAM has been proven to have good test-retest reliability (ICC range 0.758–0.970) and internal consistency (Cronbach’s alpha for FAAM-ADL and FAAM-Sport are 0.879 and 0.901, respectively) among Chinese people (González et al., 2017).

### 2.3 Ankle isokinetic muscle strength and proprioception tests

The IsoMed 2000 isokinetic dynamometer from Germany was used to test plantar flexion, dorsiflexion, inversion, and eversion of the unstable ankle in all participants (60°/s, 5 repetitions), following the methods of our previous study (Chang et al., 2024). Peak torque (PT) was selected as the test indicator, which represents the maximum output torque (Nm) generated by muscle contraction throughout the joint movement and reflects the participants’ muscle strength (Cheng et al., 2025; Cheng and Jiang, 2020). After the test, the participants maintained the test posture with the angular velocity set at 1°/s. The participants’ eyes were covered with a black cloth, and music was played through headphones. The isokinetic device moved the ankle through plantar flexion, dorsiflexion, inversion, and eversion. When participants perceived the ankle movement, they pressed a button (the device stopped), and the joint angle at that time was recorded (proprioception threshold). Each participant’s data was recorded three times, and the average value was taken. A smaller angle indicates better ankle proprioception (Chang et al., 2024).

### 2.4 Balance ability test

According to the methods of our previous study (Chang et al., 2024), the balance ability of the unstable ankle was tested. Participants stood on a three-dimensional force platform produced by KISTLER (Model: 9287B) with one foot for 10 s, repeated three times (with a 1-min interval between each test), and the average value was taken. The test indicators included the maximum displacement and average speed of the centre of pressure in the anterior-posterior (AP) and medial-lateral (ML) directions. Larger values indicate poorer balance ability (Wang et al., 2023).

### 2.5 Statistical analysis

Data were processed using SPSS 19.0 to calculate the mean ± standard deviation. The Shapiro-Wilk test was used to assess the normality of the data. Two-way ANOVA was conducted to examine the interaction between group and time. If an interaction was present, further tests were performed to determine whether there were separate effects of time or group. If no interaction was found, main effects were assessed (Cheng et al., 2025). The significance level was set at α = 0.05.

## 3 Results

**FIGURE 2:**
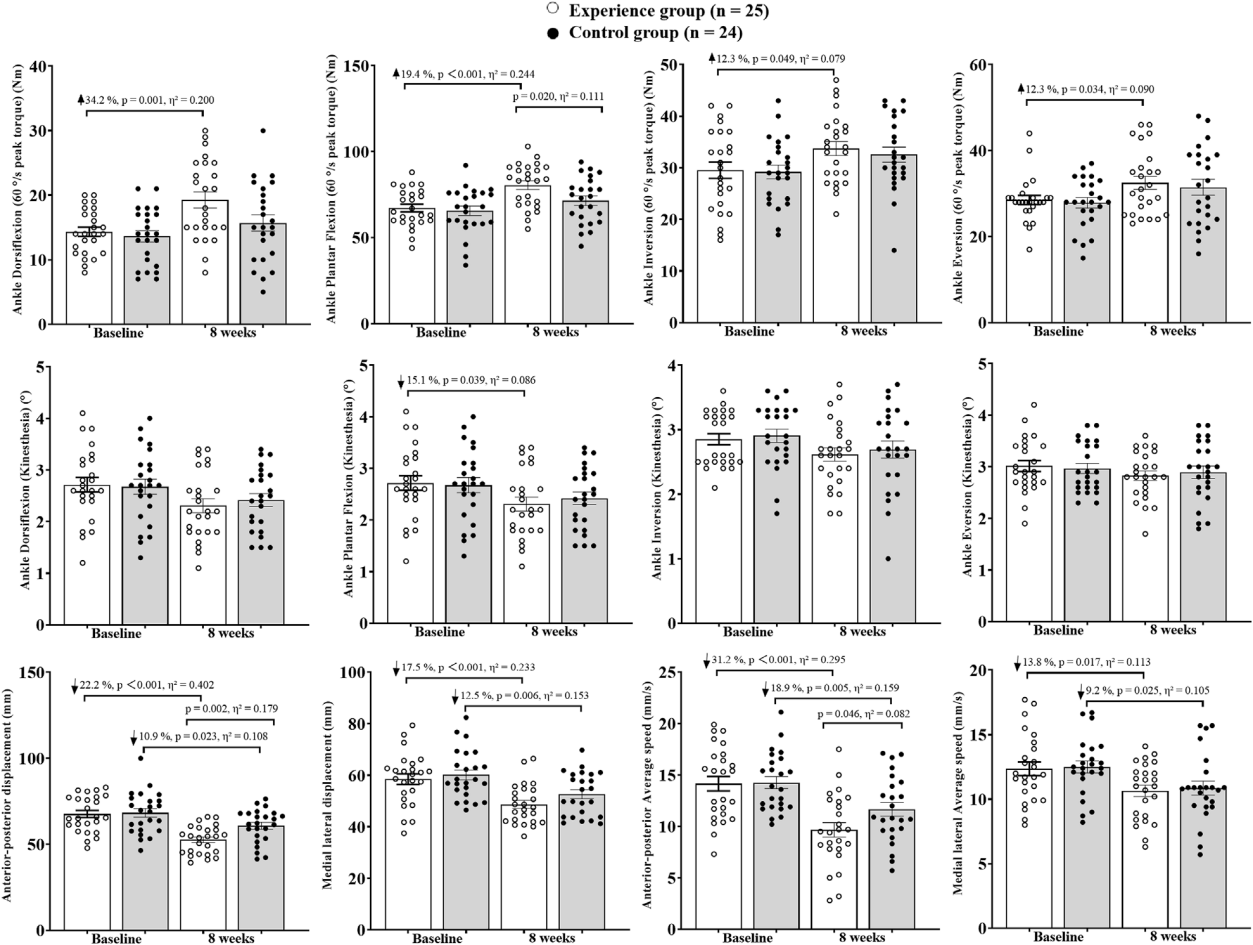
Participants had ankle muscle strength, proprioception and balance.

Firstly, the Shapiro-Wilk test confirmed that the data were normally distributed and homoscedastic. The two-way analysis of variance showed no interaction between group and time for all test indicators (*p* > 0.05). Further analysis was conducted to determine whether there were main effects of group or time. It was found that there were main effects of group for Plantar Flexion peak torque (*p* = 0.042, η^2^ = 0.043) and Anterior-posterior displacement (*p* = 0.036, η^2^ = 0.046). Only Dorsiflexion proprioception (*p* = 0.114, η^2^ = 0.026) and Eversion proprioception (*p* = 0.212, η^2^ = 0.017) showed no time effect, while all other indicators exhibited time effects (*p* < 0.05). The results are shown in Figures 1, 2.

At the 8th week, intergroup comparison revealed that the experimental group had significantly higher FAAM-ADL (*p* = 0.043, η^2^ = 0.084) and Plantar Flexion peak torque (*p* = 0.020, η^2^ = 0.111) than the control group. The Anterior-posterior displacement (*p* = 0.002, η^2^ = 0.179) and Average speed (*p* = 0.046, η^2^ = 0.082) were significantly lower in the experimental group compared to the control group. No significant differences were found between groups for other indicators (*p* > 0.05).

Intrigroup comparison between the 8th week and baseline showed that in the experimental group, CAIT increased by 16.2% (*p* = 0.006, η^2^ = 0.149), FAAM-ADL increased by 36.2% (*p* < 0.001, η^2^ = 0.378), and FAAM-Sport increased by 37.8% (*p* < 0.001, η^2^ = 0.320). The peak torque of ankle Dorsiflexion increased by 34.2% (*p* = 0.001, η^2^ = 0.200), Plantar Flexion by 19.4% (*p* < 0.001, η^2^ = 0.244), Inversion by 12.3% (*p* = 0.049, η^2^ = 0.079), and Eversion by 12.3% (*p* = 0.034, η^2^ = 0.090). The threshold of motion perception for ankle Plantar Flexion decreased by 15.1% (*p* = 0.039, η^2^ = 0.086). The Anterior-posterior displacement decreased by 22.2% (*p* < 0.001, η^2^ = 0.402) and Medial lateral displacement by 17.5% (*p* < 0.001, η^2^ = 0.233). The Average speed in the Anterior-posterior direction decreased by 31.2% (*p* < 0.001, η^2^ = 0.295) and in the Medial lateral direction by 13.8% (*p* = 0.017, η^2^ = 0.113).

In the control group, CAIT increased by 6.4% (*p* = 0.115, η^2^ = 0.053). FAAM-ADL increased by 20.9% (*p* < 0.001, η^2^ = 0.254) and FAAM-Sport by 26.3% (*p* < 0.001, η^2^ = 0.271). The Anterior-posterior displacement decreased by 10.9% (*p* = 0.023, η^2^ = 0.108) and Medial lateral displacement by 12.5% (*p* = 0.006, η^2^ = 0.153). The Average speed in the Anterior-posterior direction decreased by 18.9% (*p* = 0.005, η^2^ = 0.159) and in the Medial lateral direction by 9.2% (*p* = 0.025, η^2^ = 0.105).

## 4 Discussion

Building on previous research, this study examined whether adding WBVT to strength training and acupuncture enhances ankle stability and function. The study partially confirmed the hypothesis that adding WBVT to strength training and acupuncture further improves ankle function, Plantar Flexion strength, and anterior-posterior balance ability in patients with CAI.

### 4.1 Ankle stability and function

The results of this study show that both the control and experimental groups improved the stability and functional status of the ankle joint in patients with CAI. The experimental group’s CAIT scores increased by 16.2% from baseline, higher than the control group (6.4% increase). Additionally, the improvement in FAAM-ADL in the experimental group (η^2^ = 0.084) was approximately 1.73 times that of the control group (36.2% vs. 20.9%), suggesting that WBVT optimises neuromuscular control efficiency, further enhancing functional adaptability in daily activities. This finding extends our previous research, which showed that 8 weeks of strength training combined with acupuncture effectively improved ankle stability (Chang et al., 2024).

This may be related to strength training enhancing the structural adaptation of the ankle muscle group through progressive resistance stimulation (Smith et al., 2012), while acupuncture regulates and inhibits pain through the acupoints Taixi (KI3) and Kunlun (BL60) (Chang et al., 2024). The two interventions work synergistically to improve the stability of the ankle joint in patients with CAI. Additionally, WBVT may activate muscle spindle Ia fibres and skin/joint capsule mechanoreceptors through vibratory stimulation (Chang et al., 2021), thereby enhancing neuromuscular control efficiency in daily activities (the experimental group’s FAAM-ADL increased by 36.2%). This neuroplasticity change may accelerate the relearning process of functional movement patterns, enabling patients with CAI to exhibit better movement control in complex daily life scenarios.

### 4.2 Ankle isokinetic muscle strength and proprioception

This study found that the experimental group’s Plantar Flexion peak torque significantly increased by 19.4% compared to the control group, and Dorsiflexion peak torque increased by 34.2%. This suggests that WBVT may preferentially activate the fast-twitch muscle fibres of the gastrocnemius-soleus complex, improving neuromuscular drive efficiency and muscle fibre synchronisation (Cheng et al., 2025; Hoveidaei et al., 2024). Additionally, the study found that the experimental group showed significant improvements in peak torque for Plantar Flexion, Dorsiflexion, Inversion, and Eversion. In contrast, 8 weeks of strength training combined with acupuncture did not significantly improve peak torque in these directions in patients with CAI. This result differs from our previous study (Chang et al., 2024) and may be related to individual differences among participants. The experimental group’s acupuncture regulation of the deep peroneal nerve (Taixi acupoint) and peroneal muscle group (Kunlun acupoint), combined with the specific activation of muscle fibres by WBVT’s high-frequency vibration (Asahina et al., 2023), ultimately achieved synchronous improvement in multi-directional muscle strength. The mechanism may involve acupuncture’s neuroregulatory effects on the anterior tibialis and peroneal muscle groups (Chang et al., 2024), combined with the muscle strength enhancement induced by resistance training with elastic bands.

It is noteworthy that only the experimental group’s Plantar Flexion proprioception threshold significantly decreased by 15.1%, while no statistical differences were found in the Dorsiflexion, Inversion, and Eversion directions. This differs from previous meta-analyses (Han et al., 2022), which showed that patients with CAI primarily exhibit decreased proprioception in the Inversion/Eversion directions. This may be related to the combined intervention in this study. Firstly, during WBVT, participants maintained a Plantar Flexion position in unilateral standing, enhancing dynamic proprioception in the Plantar Flexion direction through vibratory stimulation. Secondly, acupuncture may improve the efficiency of muscle spindle afferents by regulating the gamma motor system (Kagitani et al., 2010). The gamma motor system is primarily responsible for regulating muscle spindle sensitivity, and enhancing gamma motor neuron activity can improve muscle responsiveness to stretch stimuli (Wilkinson, 2021). In this study, the combined effect of acupuncture and resistance training in Eversion may have specifically strengthened proprioception in the Eversion direction. This synergistic effect may optimise neuromuscular control and improve proprioceptive efficiency in the Eversion direction. Additionally, the introduction of the eyes-closed balance phase (weeks 5–6) further promoted proprioceptive adaptive remodelling in the Plantar Flexion position by depriving visual compensation. Although the experimental group’s Eversion peak torque increased by 12.3%, the lack of significant changes in Eversion proprioception suggests that muscle strength enhancement may primarily compensate through central motor control rather than peripheral sensory optimisation.

### 4.3 Balance ability

This study found that the experimental group’s balance improvement in the AP direction (η^2^ = 0.179) was better than that in the ML direction (η^2^ = 0.046), with an effect size difference of 3.9 times, which may be directly related to the increase in Plantar Flexion strength (19.4%, η^2^ = 0.111) and the decrease in Plantar Flexion proprioception threshold (15.1%, η^2^ = 0.086). The limited improvement in the ML direction, due to the modest increase in Inversion/Eversion strength (12.3%, η^2^ = 0.079) and no significant improvement in proprioception, indicates that the current intervention’s stimulation intensity for the Inversion/Eversion muscle group and its proprioception is insufficient, requiring further strengthening of frontal plane loading.

This may be related to the following factors. Firstly, WBVT enhances the co-activation efficiency of the Plantar Flexion-Dorsiflexion muscle group through vibratory stimulation (Hawkey and Dallaway, 2020). Combined with the 1 kg load phase, which induces adjustments in the co-activation pattern of the anterior tibialis and gastrocnemius muscles, it significantly improves the ability to counteract disturbances in the sagittal plane. Secondly, acupuncture regulates neural conduction in the Taixi (KI3) and Kunlun (BL60) acupoint areas, enhancing the coordination of muscles around the ankle joint (Chang et al., 2024). Previous studies have shown that acupuncture can simultaneously improve anterior-posterior and medial-lateral balance abilities in patients with CAI (Luan et al., 2023). However, in this study, no intergroup differences were found in the ML direction (the experimental group’s ML displacement decreased by 17.5% vs. the control group’s 12.5%). It is preliminarily speculated that the sagittal-plane-dominant stimulation of WBVT may have some impact on the potential benefits of acupuncture in the frontal plane, but the specific mechanism requires further research. This may also be related to the insufficient activation of the peroneal and posterior tibialis muscles’ structural adaptation in the Inversion/Eversion resistance training (5–15 pounds). Future research could consider adding lateral vibration or dynamic unstable surfaces to specifically strengthen ML control.

This study confirms that adding WBVT to strength training and acupuncture interventions can provide a more targeted rehabilitation plan for patients with CAI, especially for young people who need to quickly recover sagittal-plane dynamic control abilities (such as running, going up and down stairs).

The limitations of this study are as follows. The relatively small sample size, consisting only of university students, may restrict the generalisability of our findings to more diverse populations, including older adults or professional athletes. The 8-week intervention period is relatively brief, preventing us from assessing long-term efficacy and recurrence prevention. We did not use electromyography or other neurophysiological indicators, so the underlying mechanisms were not fully clarified. Due to the combined use of acupuncture, strength training, and WBVT, we cannot distinguish the independent contributions of each intervention. Additionally, the lack of blinding in this study may have introduced assessment bias, affecting the objectivity of the results. Future studies should expand sample diversity, extend the follow-up period, and incorporate multidimensional indicators, including neurophysiological measures, to more comprehensively explore the rehabilitation effects on patients with CAI. Future research should also investigate the neurophysiological mechanisms underlying the synergistic effects of WBVT and acupuncture, and strive to incorporate blinding to enhance research quality.

## 5 Conclusion

Adding WBVT to strength training and acupuncture significantly improves ankle function, plantar flexion strength, and anterior-posterior balance in CAI patients, supporting its integration into comprehensive, non-surgical rehabilitation protocols.

## Data Availability

The raw data supporting the conclusions of this article will be made available by the authors, without undue reservation.
